# The Good School Toolkit–Secondary to prevent violence against students: a pilot cluster randomised controlled trial

**DOI:** 10.1186/s12889-025-23913-8

**Published:** 2025-11-06

**Authors:** Jodie Pearlman, Mathew Amollo, Clare Tanton, John Bosco Apota, Yvonne Laruni, Janet Nakuti, Charles Opondo, Elizabeth Allen, Chris Bonell, Tvisha Nevatia, Devin Faris, Karen Devries

**Affiliations:** 1 London School of Hygiene and Tropical Medicine, London, UK; 2 AfriChild Centre, Makerere University, Kampala, Uganda; 3 Raising Voices, Kampala, Uganda

**Keywords:** Violence, Schools, Adolescents, Pilot trial, Uganda

## Abstract

**Background:**

Schools provide a unique opportunity to address multiple forms of violence against adolescents. Yet, few whole-school interventions to comprehensively address physical, sexual and emotional violence against adolescents from multiple perpetrators have been evaluated in the Global South. We report results of a pilot trial of the Good School Toolkit–Secondary (GST-S), an intervention for secondary schools in Uganda. The trial aimed to determine whether criteria for progression to a phase 3 trial were met based on pre-specified implementation and research feasibility criteria.

**Methods:**

We conducted a pilot cluster randomised controlled trial with two arms and parallel assignment. The trial was conducted in eight secondary schools, varying by faith status and urban or rural setting, randomly selected from a list of all eligible registered schools in Kampala and Wakiso Districts. Schools were randomised to control or intervention arms and aware of their allocation. The primary outcome was to determine whether criteria for progression to a phase 3 trial were met based on pre-specified criteria regarding fidelity, acceptability and understanding of GST-S, and research feasibility. Outcomes were measured using cross-sectional baseline and endline surveys among eligible school students and staff, and routine monitoring data collected during implementation.

**Results:**

Overall, seven of eight schools agreed to participate in the baseline survey, randomisation and endline survey, with three randomised to the control and four to the intervention group. The endline survey included 837 students (response rate: control, 100%; intervention, 99.4%) and 98 staff (response rate: control, 91.1%; intervention, 95.0%). There were delays to the trial due to Covid-19 and an Ebola outbreak. Despite this, all pre-specified implementation and feasibility criteria were met. The intervention was acceptable and understandable to students and staff, was delivered with fidelity, and the trial demonstrated good research feasibility.

**Conclusions:**

We present the first evidence that it is feasible to deliver a whole-school intervention aiming to address physical, sexual and emotional violence against adolescents from multiple perpetrators including peers, teachers and intimate partners in sub-Saharan Africa. Based on our results, we recommend progression to a phase 3 trial with minor refinements to the research methods and intervention.

**Trial registration:**

Pan African Clinical Trials Registry, PACTR202009826515511, 16/09/20.

**Supplementary Information:**

The online version contains supplementary material available at 10.1186/s12889-025-23913-8.

## Background

Estimates suggest that one billion children globally experience emotional, physical or sexual violence each year [1]. Evidence shows that experiencing violence in childhood increases the risk of adverse social, health and economic outcomes, including poorer physical and mental health, sexual risk behaviours, behaviour disorders, substance abuse, lower educational attainment, worse employment outcomes and perpetration of violence [2–7]. Ending violence against children is an important part of the 2030 Sustainable Development Goals, where it is included as a specific target and in relation to goals for quality education and gender equality [8].

In Uganda, a national survey in 2015 found that over three-quarters of young men and women reported experiencing at least one type of violence during childhood. The survey found that 59% of young women and 68% of young men reported experience of physical violence, 34% of young women and 36% young men reported experience of emotional violence, and 35% of young women and 17% of young men reported experience of sexual violence during their childhoods [9]. The survey also found that teachers, peers and romantic partners were common perpetrators of violence, all of whom are likely to interact with students in schools. Ending violence against children is a key priority area within the Uganda National Child Policy [10]. Yet, analysis of the Ugandan government’s budget allocation showed that the child protection sector is not being allocated sufficient funds despite identification of child protection as a national priority [11].

Despite the burden of violence and commitment to reducing it, there is a lack of effective interventions aiming to address violence in adolescence that have been evaluated in the Global South [12–14]. Most interventions aiming to prevent violence against adolescents in the Global South aim to address only one type of violence, most commonly violence from peers [15–17]. Very few interventions aim to address physical, sexual or emotional violence perpetrated by teachers towards students [18], and only three interventions that aim to reduce both teacher and peer violence against children have been evaluated in randomised controlled trials and shown to be effective in the Global South [19–21]. However, these were all in pre-primary or primary schools, with limited evidence for interventions in secondary schools.

Schools provide a unique opportunity to address multiple forms of violence against adolescents and the World Health Organisation (WHO) recommends whole-school approaches to prevent violence [22, 23]. By bringing together a range of important individuals in an adolescent’s life, including teachers, peers, administrative staff, parents and the community, these approaches have the potential to reduce multiple forms of violence that adolescents may experience.

The Good School Toolkit (GST) is a whole-school approach to prevent violence that works by influencing the operational culture of a school. It was developed by Ugandan non-governmental organisation Raising Voices, originally for primary schools. A randomised controlled trial (RCT) of the GST for primary schools (GST-P) in 2012-14 showed that GST-P led to a 42% reduction in reported past week physical violence from staff to students, in addition to reducing severe physical violence and injury from staff to students, and physical and emotional violence from peers [20]. The WHO INSPIRE guidelines highlight the GST-P as an evidence based violence prevention programme [24]. Following its success in primary schools, Raising Voices has since adapted the GST-P for use in secondary schools [25]. This paper describes the results of a pilot RCT of the Good School Toolkit–Secondary (GST-S).

### Aims, objectives and research questions

The primary aim of this pilot trial was to determine whether criteria for progression to a phase 3 trial were met in terms of pre-specified acceptability and feasibility criteria. In this paper, we report on the following objectives and research questions [26]:

Objective 1: To refine and finalise the GST-S intervention, ensuring acceptability and understanding.

- Is the intervention acceptable and understandable to male and female students of all ages and staff?

Objective 2: To understand the feasibility of delivery of the intervention.

- Is it feasible to implement the GST-S with fidelity over 18 months?
- Which activities do schools implement?
- What is the pace of implementation and intensity of different activities?

Objective 3: To explore design parameters for a subsequent phase 3 trial.

- For sample size calculations: What are preliminary estimates and 95% confidence intervals for prevalence and clustering of phase 3 trial primary outcomes, adolescent intimate relationships, and effectiveness of the intervention?
- For recruitment and retention: Are secondary schools willing to be recruited and randomised, and will both intervention and control schools remain in a trial? What are individual student response rates? What are levels of school attrition over time, particularly in control schools?

In a separate qualitative paper, we report further on acceptability and understanding of new content, coherence of GST-S following introduction of the new activities and concepts (detailed in Additional File 2), and the need for refinements to the intervention.

## Methods

### Design

This study is a pilot cluster randomised controlled trial with two arms and parallel assignment. A cluster design was chosen given the intervention is implemented at school level. Cross-sectional baseline and endline surveys (unlinked at individual level) were conducted in schools in March 2020 and July 2023, respectively, and routine monitoring data collected throughout implementation. Qualitative data were collected throughout implementation and the results will be published separately.

This trial was registered at the Pan African Clinical Trials Registry (PACTR202009826515511) and the protocol for the study has been published [26]. This paper presents the main quantitative pilot trial results.

### Setting

This study took place in Kampala and Wakiso Districts, Uganda. These districts have populations of almost 2 million and almost 3.5 million, respectively [27]. Both areas have higher income levels than the rest of Uganda, but also substantial inequities in incomes. Kampala has some very poor and densely populated urban areas, and both districts also have rural areas which draw school populations from large catchment areas.

### Study population and recruitment

Secondary schools in Kampala and Wakiso districts were eligible if they had more than 500 students of which more than 50% were day students. Only mixed-sex schools were included to ensure adequate numbers of students in intimate partner and sexual relationships to allow exploration of new GST material on adolescent intimate partner relationships. We excluded schools that were involved in intervention studies.

Eligible schools were stratified by factors hypothesised to affect acceptability and feasibility of delivery, including whether schools were faith- or non-faith-based and located in urban or rural settings. Of all eligible schools, 20 were randomly selected across the four strata, and two schools randomly selected within each stratum. Recruitment involved preliminary visits to schools followed by meetings with headteachers about GST-S and the trial. A member of the research team obtained consent from the headteacher for the school to participate. If schools declined to participate, the next school listed within that stratum was selected.

All students enrolled at the school were eligible for participation in surveys, except if they: were unable to speak or read English or Luganda; were unable to provide informed consent; or had a significant impairment precluding questionnaire completion. Interviewers assessed language ability. All staff employed at the participating school were eligible to participate.

### Sample size

Eight secondary schools were randomly selected out of a list of all eligible schools in Kampala and Wakiso Districts. This number was large enough to allow different types of schools to be included but small enough to allow Raising Voices to support implementation in each intervention school.

For baseline and endline surveys, we aimed to randomly select 120 students in each school, stratified by grade (Senior 1 to 6) and sex from all enrolled students in those classes. The target sample size for staff was 25–30.

### Data collection and interview procedures

Baseline surveys were conducted prior to randomisation, and the endline survey 18 months after the start of implementation, all via face-to-face interviews. Interviewers received three weeks of training. All interviews were conducted in private locations which could not be overheard by students or staff, and interviewers confirmed with participants that they were happy proceeding at the agreed time and location. Interviewers went through the informed consent procedure with individual participants prior to starting the interview. This included informing the participant that we could refer them to a counsellor at a local service if they felt they would like to speak to someone following the interview. The interviewer paused the interview to check in with the participant if anyone moved nearby during the interview or the interviewee appeared uncomfortable. Interviewers stopped interviews if a participant was distressed and referred participants for support where appropriate.

Referrals for participants were made based on pre-defined criteria, using similar processes to the GST-P trial but adapted to adolescent needs. Students were referred to local child protection partners according to the type, severity and timeframe of violence exposure disclosed, and severity of mental health problems. All students participating in the study were offered the opportunity to receive counselling regardless of experiences disclosed. Adults participating in the study were also provided with a list of local organisations which could be contacted for support, though this was optional for adults. Full referral processes are detailed in the published protocol [26].

Participants were able to complete the survey in English or Luganda. We cognitively tested new measures on acceptability and understanding prior to endline data collection to ensure that staff and students understood the questions, and refined questions based on feedback. Final questionnaires for baseline and endline were pilot tested to check the length and flow.

We randomised students and staff to complete a subset of questions using audio-computer-assisted self-interviewing (ACASI) rather than face-to-face interviews (FTFI). The results of this sub-study will be published separately (registered at the Pan African Clinical Trials Registry, PACTR202311533798407).

### Allocation

Following the baseline survey, the headteachers of each school were invited to a meeting to allocate their school to intervention or control. For each stratum of schools, headteachers placed their school names into an opaque bag. One headteacher from each pair drew names out of the bag, with schools allocated to intervention or control conditions according to the order in which they were drawn out of the bag. The study was unblinded, with schools aware of their allocation to either control or intervention condition.

### Intervention and control conditions

Schools in the intervention arm received GST-S plus ‘usual care’ provided by schools, which currently does not include specific lessons or programming related to violence prevention. Control schools received usual care during the study and the GST-S after the endline survey.

#### The GST-S

Developed by Ugandan NGO Raising Voices, the GST is a whole-school complex behavioural change intervention aiming to change the overall operational culture of a school and prevent violence against children. The intervention itself draws on the Transtheoretical Model of Behaviour Change [28]​​, supporting schools to work through the process of change by participating in six steps over 18 months. This aims to bring about change in the school operational culture through four entry points: (1) improving the relationship between students and teachers; (2) improving peer relationships; (3) promoting student and teacher participation and connectedness to school; and (4) strengthening engagement from caregivers and local communities in school ​ [29]. The intervention also contains a range of behaviour change techniques identified in the COM-B model [30, 31] as successful ingredients in other areas of behaviour change, such as setting goals, creating action plans and modelling desired behaviours. Raising Voices has developed a programmatic theory of change to describe this process [29], and our previous work has identified the formation of improved student-teacher relationships as a key element driving change [29]. It differs from other interventions trialled to reduce teacher violence in that it is not curriculum-based [32], and it takes a whole-school approach and engages multiple actors rather than focusing only on teachers [18, 33, 34].

The programme is led by at least two teacher and two student ‘protagonists’ in each school, in addition to community members affiliated with the school, and supported by a trained violence prevention advocate from Raising Voices. Schools can choose to implement any of around 60 different activities and core structures. These draw on ideas such as improving the learning environment, using positive discipline as opposed to physical discipline methods, and understanding power dynamics in relationships which are thought to underpin multiple types of violence. Schools are provided with materials, including handbooks that describe activities, posters to put up in school and cartoon booklets. These are all publicly available at www.raisingvoices.org and more detailed information has been published previously [20].

The GST was originally trialled in primary schools and has been adapted [25] to address the unique needs of adolescents. The GST-S includes strengthened content and new activities on gender equality, peer violence and relationship values, and sexual violence and power in relation to sexuality and transactional relationships. Other minor changes include adaptation or removal of activities based on their effectiveness at primary school level and language changes to more appropriately reflect secondary school level. A table summarising the new and adapted content in GST-S compared to GST-P is included in the trial protocol [26] and Additional File 2.

### Outcomes

#### Primary outcome

The primary outcome was whether criteria for progression to a phase 3 trial were met. Criteria were judged against a traffic-light system to determine progression (Table 1). An overall green classification indicated progression to a phase 3 trial, amber as progression with plans to address specific issues, and red as no progression and no further feasibility work. Specific criteria making up the overall judgement are included in Table 2.

**Table 1:** Overall categories for progression

Overall judgement	Criteria (with respect to outcomes in Table 2)
Green – OK to progress	- No more than 1 amber rating for outcomes concerning ‘Intervention implementation’ and ‘Research feasibility’ - No red ratings
Amber – progress with plans in place to address specific issues	- No more than 2 amber ratings for outcomes concerning ‘Intervention implementation’ and ‘Research feasibility’ - No more than 1 red rating
Red – no progression without further feasibility work	- More than 2 amber ratings for ‘Research feasibility’ - More than 1 red rating

Previously published in the GST-S protocol [26]. Specific criteria related to outcomes are included in Table 2

**Table 2:** Specific criteria for progression

		Definition for categorisation of progression criteria		
Type of outcome	Topic	Green	Amber	Red
Intervention implementation (intervention schools)	Acceptability	*Per item*: ≥ 80% report acceptability *Overall acceptability*: ≤2 red items (no red items in core dimensions*)	*Per item*: ≥ 70% report acceptability *Overall acceptability*: ≤ 3 red items (no red items in core dimensions)	*Per item*: < 70% report acceptability *Overall acceptability*: > 3 red items overall, or any within core dimensions
	Understanding	*Per item*: ≥ 80% report understanding *Overall understanding*: ≤3 red items (no red items in core dimensions)	*Per item*: ≥ 70% report understanding *Overall understanding*: ≤4 red items (no red items in core dimensions)	*Per item*: < 70% report understanding *Overall understanding*: >4 red items overall, or any within core dimensions
	Fidelity	≥ 3 of 4 intervention schools implement with fidelity at endline Fidelity defined as ≥50% of core fidelity markers in place	N/A	< 3 of 4 intervention schools implement with fidelity at endline Fidelity defined as ≥50% of core fidelity markers in place
Research feasibility (all schools)	Enrolment	≥ 6 of 8 schools agree to participate in the baseline survey	5 of 8 schools agree to participate in the baseline survey	< 5 of 8 schools agree to participate in the baseline survey
	Randomisation	≥ 6 of 8 schools participating in the baseline survey agree to be randomised	5 of 8 schools participating in the baseline survey agree to be randomised	< 5 of 8 schools participating in the baseline survey agree to be randomised
	Follow-up	≥ 6 of 8 schools randomised agree to endline data collection	5 of 8 schools randomised agree to endline data collection	< 5 of 8 schools randomised agree to endline data collection
	Response rate	> 70% of eligible students respond to surveys	> 60% of eligible students respond to survey	< 60% of eligible students respond to survey

Adapted from a table previously published in protocol [26]

*Core dimensions for acceptability include “General acceptability and affective attitude” and “Burden”. Core dimensions for understanding include all items related to violence: violence against children, positive discipline, sexual harassment, peer violence

#### Acceptability and understanding measures

To measure acceptability, we developed a series of questions (7 for students, 9 for staff) to capture the multiple dimensions of the concept, including general acceptability and affective attitude, burden, ethicality, and self-efficacy [35]. To measure understanding, we developed questions (13 for students, 15 for staff) to measure understanding of core concepts communicated through GST-S, such as violence, power, positive discipline, gender and mental health. These items are listed in Additional File 3.

For the acceptability and understanding constructs, each item was classified as red, amber or green based on the number of individuals strongly agreeing or agreeing to the statement (or strongly disagreeing/disagreeing for statements phrased in the direction that implies a negative acceptability or understanding). Red was classified as < 70% agreeing/strongly agreeing, amber as ≥ 70% and < 80% agreeing/strongly agreeing, and green as ≥ 80% agreeing/strongly agreeing. Items were classified individually to allow identification of any questions that may not have worked in measuring acceptability or understanding. When considering acceptability in relation to overall trial progression, we decided a priori to tolerate no more than two items being classified as red, and all items within “General acceptability and affective attitude” and “Burden” being classified as green or amber given their relative importance according to expert opinion from implementers at Raising Voices. When looking at overall understanding, we decided a priori to tolerate no more than three items being classified as red (given the additional number of questions compared to acceptability), and all concepts related to violence must be classified as green or amber given that violence is the core outcome of the GST-S.

#### Fidelity measures

Our original progression criteria for fidelity was defined as implementation of ≥ 50% of 21 new GST-S activities plus core structures in place, as measured by the action plans completed by schools as part of their implementation. However, early on, we realised that secondary schools were not completing their written action plans, despite site visits from Raising Voices confirming that implementation was occurring.

We therefore conducted a focus group discussion with Raising Voices and identified eleven markers across the GST-S steps that were important for fidelity (Table 3) and could be assessed using routine data collected by Raising Voices during their implementation. At least half of these markers were deemed necessary for successful implementation. Data came mainly from a standardised scorecard measuring key outcomes of implementation at school-level, completed by a Raising Voices staff member or resource person (trained and supported by Raising Voices to implement GST-S) by conducting observations within schools and interacting with students and teachers.

**Table 3:** Markers of fidelity across GST-S steps

GST-S step​	Proposed structures/activities​
Step One: Your team and network​	• Existence of GST-S teacher committee • ​Existence of GST-S student committee • Existence of GST-S parent committee
Step Two: Preparing for change​	• Implemented at least 2 activities • Held committee meeting at least once a month​
Step Three: Good teachers and teaching​	• Teacher-student meetings held • Feedback to teachers provided​
Step Four: Positive discipline​	• Existence of students’ court • Existence of wall of fame​
Step Five: Good learning environment​	• At least one activity/structure promoting student’s voice e.g. functional suggestion boxes; existing policies promoting learners’ safety​
Step Six: Administration and future​	• Sustainability plan in place​

Implementation of GST-S was further explored by looking at the support visits provided by Raising Voices to schools, including the number, type and participants.

We also measured individual-level exposure to GST-S using 13 survey questions adapted from those used in GST-P. These contained questions that reflected both active and passive participation, and some of the new content in GST-S. Items are included in Additional File 6.

#### Trial design parameters

To assess research feasibility, we kept records of schools approached to measure number of schools agreeing to participate in the baseline and endline surveys, be randomised, and the response rate of students and staff.

Surveys included questions on the experience of physical, sexual and emotional violence from school staff, peers and intimate partners in the past year, term and week. This was self-reported by students using a modified version of the International Society for the Prevention of Child Abuse and Neglect Child Abuse Screening Tool-Child Institutional tool [20, 36]. Participants reported violence either via a FTFI, or via an ACASI.

### Statistical analysis

Given that this was a pilot study, analysis of primary outcomes was mainly descriptive. This included tabulation of proportions for each dimension within the progression criteria. For acceptability and understanding, we tabulated percentages of individuals agreeing/strongly agreeing and disagreeing/strongly disagreeing for each item. We conducted an exploratory subgroup analysis to look at differences in acceptability and understanding by sex, being in lower vs. upper school, and number of meals eaten yesterday (as a proxy for socio-economic status). To do this, we estimated the risk difference (RD) with 95% CIs and *p*-values using a binomial regression model adjusting for clustering by school.

For other outcomes, we tabulated descriptive summaries of baseline and follow-up data by intervention arm. In line with good practice, we did not perform significance tests to test for differences at baseline [37]. We conducted an exploratory intention-to-treat analysis for the analysis of indicative phase 3 trial outcomes. Analyses accounted for clustering by school and were adjusted for sex, assignment to report violence exposure via ACASI or FTFI, urban/rural school, faith/non-faith-based school, and the baseline school-level mean of the outcome. For individuals where baseline data were missing, analyses were adjusted for the mean baseline value of the outcome for that arm. We present 95% confidence intervals estimates for the effect of GST-S on outcomes rather than point estimates for the OR given that this is a pilot trial. We also present the ICC for the main phase 3 outcomes.

All analyses were carried out in Stata 18.

### Deviations to the protocol

The pilot trial was severely affected by both the Covid-19 pandemic and prolonged school closures from 2020 to 2022, and an Ebola outbreak in Uganda from September 2022 to January 2023, resulting in several deviations from our original protocol. We were partway through our baseline data collection in schools in March 2020 when Covid-19 resulted in immediate school closures and subsequent national lockdowns. Subsequent closure of schools for two years delayed implementation of the intervention. Implementation re-started in January 2022 when schools re-opened but was slower than anticipated due to competing pressures in schools. The Ebola outbreak led to additional school closures, and reduction of Raising Voices activity in schools and communities. Schools were behind with implementation given the closures and their competing demands, and we delayed collection of data for the endline survey from March to July 2023.

During baseline data collection, we reduced the number of teachers sampled at endline from 20 to 15 per school (total of 120) due to fewer numbers of teachers in schools than expected.

To measure fidelity, we had planned to use data from structured observations to estimate the total number of activities implemented in each school, based on planned activities and those observed during 32 random observations, randomly selected from action plans developed by schools. However, as detailed above, secondary schools were not completing their written action plans, despite site visits from Raising Voices confirming that implementation was occurring. We were thus unable to randomly select activities to observe. We were instead guided by expert opinion from Raising Voices on eleven key markers of fidelity that could be measured using their routinely collected data.

The GST is designed to be a flexible programme tailored to schools’ needs, and during implementation, Raising Voices made minor adaptations to the GST-S based on experiences during implementation and emerging feedback from schools. These included increasing the numbers of students on the committees depending on the size of the school; and breaking down activities into smaller more frequent chunks to allow better integration into the school timetable.

While we intended to analyse data blind to treatment allocation, this was not possible given the differing number of schools in control and intervention arms, of which the researcher analysing the data was aware.

### Ethics

This study was approved by the London School of Hygiene & Tropical Medicine Ethics Committee, the Mildmay Uganda Research Ethics Committee, and the Uganda National Council for Science and Technology. Any adverse events related to the intervention were monitored during routine visits to schools but the main risks to participants concerned participation in the research process and need for support following disclosure of violence or mental health problems during data collection.

### Role of the funding source

The funders played no role in the design of the study, data collection, analysis, interpretation or preparation of the manuscript.

## Results

### Participation, randomisation and follow-up

Recruitment started in November 2019. Of 225 eligible schools, five of the original eight schools agreed to participate. Two of the original eight declined to participate due to busy school schedules. The first two reserve schools also declined for reasons linked to their internal processes. The first required internal approval from multiple administrative levels, and the second we replaced given that they required the board to approve participation and no date was set for the board to sit. The next two schools approached agreed to participate. Eight schools were fully recruited by February 2020, of which six had been originally selected. Following headteacher consent, one of the original schools declined to participate in the baseline survey, randomisation or endline survey as this school required the Parent Teacher Association to ascertain the authenticity and safety of GST-S to students.

Progression criteria for enrolment was amber, with five of eight schools originally approached agreeing to participate in the baseline survey. Including those replaced, seven of eight schools agreed to participate in the baseline survey conducted in March 2020. Only 386 students (4 schools) and 85 staff (5 schools) participated in the baseline survey due to the Covid-19 pandemic which led to closure of schools during baseline data collection. In schools that did participate at baseline, target sample sizes were not reached and reasons for individuals not participating was not known for all individuals due to sudden school closures. It was decided to proceed with the trial despite missing baseline data given its primary focus on acceptability, understanding and fidelity rather than effectiveness.

**Fig. 1:**
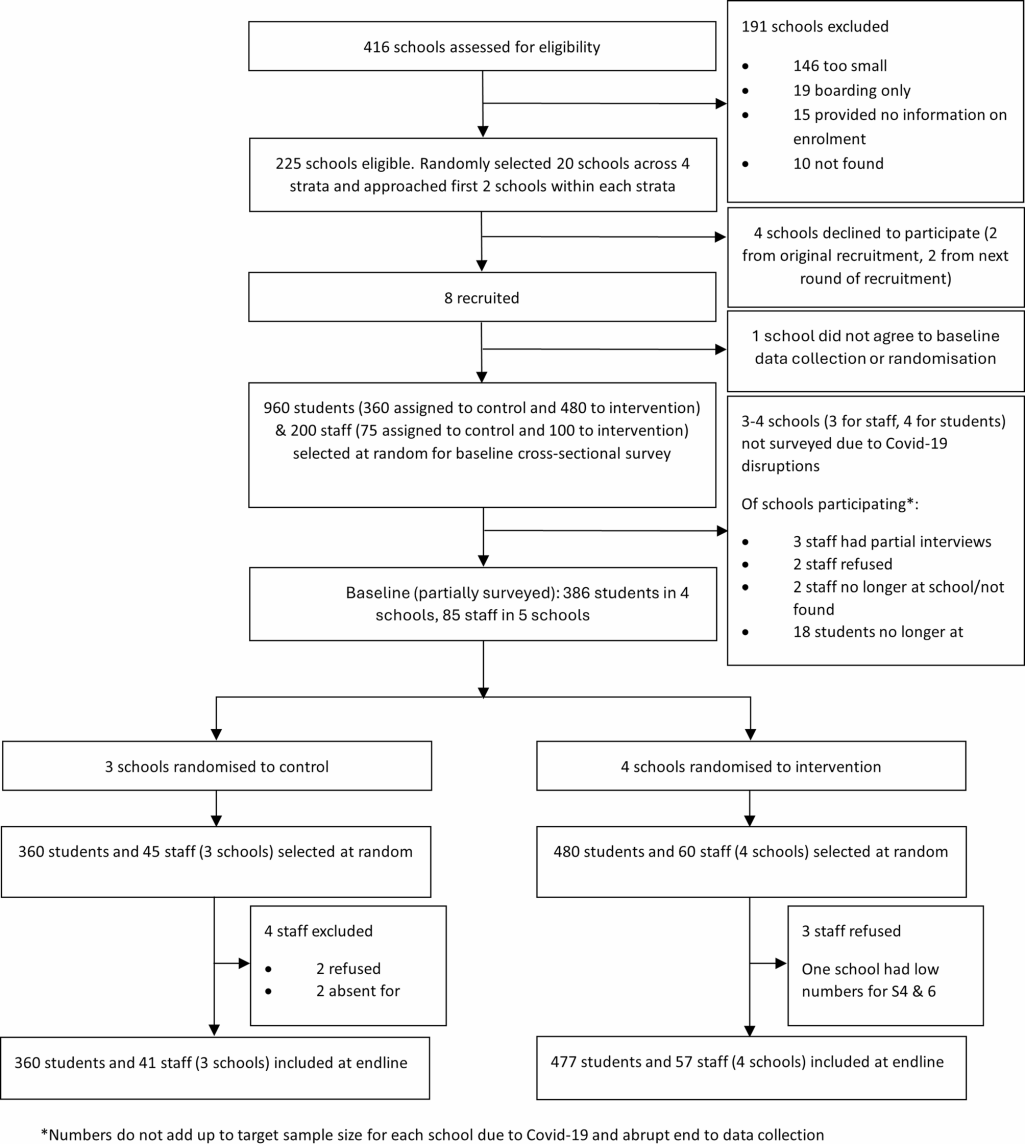
Trial flow diagram

Our progression criteria for both randomisation and follow-up were green, with all seven schools that participated in the baseline survey agreeing to randomisation to intervention or control, and to participation in the endline survey. Overall, 837 students (response rate: 100.0% in control arm, 99.4% in intervention arm) and 98 staff (response rate: 91.1% in control arm, 95.0% in intervention arm) participated in the endline survey. Criteria for progression around response rate were based on endline survey response rates given that we could not follow up all individuals or schools in the baseline survey. Details of the numbers of students, staff and schools participating at baseline, randomisation and follow up are described in the CONSORT diagram (Fig. 1).

While contamination between arms was possible, for example if headteachers from control and intervention schools engaged with each other as was the case in the GST-P trial [20], we did not observe any instances of this during the trial.

### Baseline characteristics of staff and students

Demographic characteristics of students and staff at baseline are presented in Table 4. Intervention and control student participants had a mean age of 15.9 and 16.2 years, and were 50.2% and 56.6% female, respectively. Participants were approximately evenly distributed across school classes, with the exception of S5. For staff participating in the baseline survey, mean age was 39.75 years and 37.53 years, and 33.3% and 42.9% of participants were female in intervention and control schools, respectively. It is important to note the missing data at baseline which may partly explain differences between arms. Characteristics of staff and student characteristics at endline are also included in Table 5. Different staff and students (within the same schools) were surveyed at endline.

**Table 4:** Characteristics of students and staff at baseline

			Control		Intervention		All schools	
			n	%	n	%	n	%
**Student characteristics**								
Number of students			99	25.7%	287	74.4%	386	100.0%
Sex								
Male			43	43.4%	143	49.8%	186	48.2%
Female			56	56.6%	144	50.2%	200	51.8%
Age, years (mean, SD)			16.2	2.0	15.9	2.0	16.0	2.0
School class								
S1			20	20.2%	60	20.9%	80	20.7%
S2			18	18.2%	63	22.0%	81	21.0%
S3			18	18.2%	60	20.9%	78	20.2%
S4			21	21.2%	54	18.8%	75	19.4%
S5			0	0.0%	0	0.0%	0	0.0%
S6			22	22.2%	50	17.4%	72	18.7%
Meals eaten yesterday*								
0 meals			1	1.0%	2	0.7%	3	0.8%
1 meal			15	15.2%	21	7.3%	36	9.4%
2 meals			29	29.3%	111	38.8%	140	36.4%
3 + meals			54	54.6%	152	53.2%	206	53.5%
Mode of transport to school*								
Car			5	5.3%	3	1.2%	8	2.3%
Matatu			24	25.5%	26	10.0%	50	14.1%
Walking alone			33	35.1%	51	19.5%	84	23.7%
Walking with the person I know			18	19.2%	78	29.9%	96	27.0%
Bicycle			1	1.1%	2	0.8%	3	0.9%
Sleep at school			11	11.7%	89	34.1%	100	28.2%
Boda boda			1	1.1%	12	4.6%	13	3.7%
Other			1	1.1%	0	0.0%	1	0.3%
Hours of work outside of school each day								
< 1 h			32	32.3%	76	26.5%	108	28.0%
1–2 hrs			39	39.4%	87	30.3%	126	32.6%
2–3 hrs			7	7.1%	33	11.5%	40	10.4%
3–4 hrs			6	6.1%	20	7.0%	26	6.7%
5 + hrs			2	2.0%	5	1.7%	7	1.8%
N/A			13	13.1%	66	23.0%	79	20.5%
Orphan*								
Not an orphan			36	81.8%	125	80.7%	161	80.9%
Single orphan			8	18.2%	26	16.8%	34	17.1%
Double orphan			0	0.0%	4	2.6%	4	2.0%
Functional difficulty			55	55.6%	128	44.6%	183	47.4%
Absence from school in previous week			15	15.2%	33	11.5%	48	12.4%
**Past week violence**								
Any past week violence								
From school staff			54	54.5%	109	38.0%	164	42.2%
From peers			56	56.7%	149	51.9%	205	53.1%
Physical violence								
From school staff			45	45.5%	95	33.1%	140	36.3%
From peers			23	23.2%	43	18.8%	77	20.0%
Sexual violence								
From school staff			2	2.0%	5	1.7%	7	1.8%
From peers			10	10.1%	29	10.1%	39	10.1%
Emotional violence								
From school staff			29	29.3%	50	17.4%	79	20.5%
From peers			49	49.5%	137	47.7%	186	48.2%
**Staff characteristics**								
Number of staff			49	49.0%	51	51.0%	100	100.0%
Age, years (mean, SD)			37.53	8.85	39.75	11.40	38.66	10.24
Sex								
Male			28	57.1%	34	66.7%	62	62.0%
Female			21	42.9%	17	33.3%	38	38.0%
Marital status								
Single			12	24.5%	11	21.6%	23	23.0%
In a relationship			1	2.0%	1	2.0%	2	2.0%
Married/living together			32	65.3%	39	76.5%	71	71.0%
Divorced/widowed			4	8.2%	0	0.0%	4	4.0%
Has children			38	79.2%	39	65.5%	77	77.8%
Housing								
Own			22	44.9%	27	52.9%	49	49.0%
Rented			7	14.3%	17	33.3%	24	24.0%
Live somewhere without paying			5	10.2%	1	2.0%	6	6.0%
Employer pays			15	30.6%	6	11.8%	21	21.0%
Meals eaten yesterday								
1 meal			6	12.2%	3	5.9%	9	9.0%
2 meals			9	18.4%	9	17.7%	18	18.0%
3 + meals			34	69.4%	39	76.5%	73	73.0%
Years in school								
0–2 years			13	26.5%	18	35.3%	31	31.0%
3–5 years			12	24.5%	12	23.5%	24	24.0%
5 + years			24	49.0%	21	41.2%	45	45.0%
Main role at school								
Teacher			48	98.0%	47	92.2%	95	95.0%
Headteacher/Deputy			0	0.0%	1	2.0%	1	1.0%
Other			1	2.0%	3	5.9%	4	4.0%

*Missing data: Students’ meals eaten yesterday: *n* = 1, students’ orphan status: *n* = 187, student mode of transport to school: *n* = 31

**Table 5:** Characteristics of students and staff at endline

Characteristic		Control			Intervention			All	
		*n*	%		*n*	%		*n*	%
**Student characteristics**									
Number of students		360	43.0%		477	57.0%		837	100%
Age, years (mean, SD)		17.13	2.04		17.28	2.18		17.22	2.12
Sex									
Male		182	50.6%		238	49.9%		420	50.2%
Female		178	49.4%		239	50.1%		417	49.8%
School class*									
S1		59	16.4%		82	17.7%		141	17.1%
S2		58	16.1%		76	16.4%		134	16.3%
S3		63	17.5%		74	16.0%		137	16.6%
S4		57	15.8%		79	17.0%		136	16.5%
S5		60	16.7%		80	17.2%		140	17.0%
S6		63	17.5%		73	15.7%		136	16.5%
Attended previous class at this school		264	73.3%		320	67.1%		584	69.8%
Meals eaten yesterday									
0 or 1 meal		20	5.6%		51	10.7%		71	8.5%
2 meals		115	31.9%		141	29.6%		256	30.6%
3 + meals		225	62.5%		285	59.8%		510	60.9%
Hours of work outside of school each day*									
< 1 hr		56	15.6%		51	10.8%		107	12.9%
1–2 hrs		101	28.1%		169	35.8%		270	32.5%
2–3 hrs		75	20.9%		94	19.9%		169	20.3%
3–4 hrs		63	17.6%		57	12.1%		120	14.4%
5 + hrs		64	17.8%		101	21.4%		165	19.9%
Orphan*									
Not an orphan		293	82.5%		381	80.6%		674	81.4%
Single orphan		56	15.8%		84	17.8%		140	16.9%
Double orphan		6	1.7%		8	1.7%		14	1.7%
Living with mother		239	66.4%		256	53.7%		495	59.1%
Living with father		169	46.9%		225	47.2%		394	47.1%
Functional Difficulty		176	48.9%		255	53.5%		431	51.5%
Mode of transport to school*									
Car		6	1.7%		2	0.4%		8	1.0%
Matatu		31	8.7%		12	2.5%		43	5.2%
Walk alone		82	22.9%		153	32.1%		235	28.1%
Walk with someone you know		40	11.2%		81	17.0%		121	14.5%
Bicycle		10	2.8%		15	3.1%		25	3.0%
Board at school		161	45.0%		167	35.0%		328	39.3%
Boda boda		28	7.8%		46	9.6%		74	8.9%
Other		0	0.0%		1	0.2%		1	0.1%
Time to get to school (if not boarding), minutes									
< 1 hr		179	49.7%		246	51.6%		425	50.8%
1–2 hrs		18	5.0%		59	12.4%		77	9.2%
2 + hrs		163	45.3%		172	36.1%		335	40.0%
Absence from school in previous week									
1 or more days missed		68	18.9%		90	18.9%		158	18.9%
**Staff characteristics**									
Number of staff		41	41.8%		57	58.2%		98	100.0%
Age, years (mean, SD)		40.4	13.2		38.0	10.1		39.0	11.5
Sex									
Male		20	48.8%		29	50.9%		49	50.0%
Female		21	51.2%		28	49.1%		49	50.0%
Marital status*									
Single		14	35.0%		9	15.8%		23	23.7%
In a relationship		1	2.5%		1	1.8%		2	2.1%
Married/living together		25	62.5%		45	79.0%		70	72.2%
Widowed		0	0.0%		2	3.5%		2	2.1%
Has children*		33	84.6%		46	82.1%		79	83.2%
Housing*									
Own		18	45.0%		24	42.1%		42	43.3%
Rented		12	30.0%		17	29.8%		29	29.9%
Live somewhere without paying		6	15.0%		4	7.0%		10	10.3%
Employer pays		4	10.0%		11	19.3%		15	15.5%
Other		0	0.0%		1	1.8%		1	1.0%
Meals eaten yesterday									
No meals		1	2.4%		1	1.8%		2	2.0%
1 meal		2	4.9%		3	5.3%		5	5.1%
2 meals		7	17.1%		6	10.5%		13	13.3%
3 + meals		31	75.6%		47	82.5%		78	79.6%
Years in school									
0–2 years		9	22.0%		19	33.3%		28	28.6%
3–5 years		9	22.0%		11	19.3%		20	20.4%
5 + years		23	56.1%		27	47.4%		50	51.0%
Main role at school									
Teacher		38	92.7%		51	89.5%		89	90.8%
Headteacher/Deputy Headteacher		1	2.4%		2	3.5%		3	3.1%
Senior Man/Woman Teacher		1	2.4%		2	3.5%		3	3.1%
Other		1	2.4%		2	3.5%		3	3.1%

*Missing data: Students: school class, *n* = 13; hours of work outside of school each day, *n* = 6; orphan, *n* = 9; mode of transport to school, *n* = 4; Staff: marital status, *n* = 2; has children, *n* = 3; housing, *n* = 1

### Acceptability, understanding and fidelity

#### Acceptability

The progression criterion for overall acceptability was classified as green. Among students in intervention schools, six items within the acceptability construct were classified as green and one as amber. Access to Good School materials (within self-efficacy) was classified as amber. Exploratory subgroup analyses suggested similar results between male and female students. Some differences were found between lower (classes S1-S4) and upper school students (classes S5-S6). More upper school students reported that students should be contributing to making decisions in school compared to lower school students (RD 8.7%, 95CI% 3.4—13.9, *p* = 0.001). Upper school students also felt more confident in taking part in GST-S activities (RD 2.7%, 95%CI 1.1—4.3, *p* = 0.001). General acceptability also differed by number of meals eaten yesterday (overall *p* = 0.010), where it was higher among students who ate two meals (RD 2.9%, 95%CI −0.4—6.3) and zero or one meals (RD 0.7%, 95%CI −4.7—6.2) compared to three or more.

For acceptability of GST-S among staff, seven of nine items were classified as green. The other two, both within the self-efficacy dimension, were red. These related to accessing Good School materials (54.4%) and receiving enough training (36.8%).

#### Understanding

The progression criterion for overall understanding was classified as green. For understanding of GST-S among students, eight items were classified as green, three as amber and two as red. Both red items were questions related to understanding of the student court. Exploratory subgroup analyses suggested similar results between male and female students with the exception of understanding of rights and shared rights, which was higher among females for both items (item 1: RD 5.7%, 95%CI 0—11.3, *p* = 0.05; item 2: RD 2.7%, 95%CI 0.7—4.7, *p* = 0.008). Results also suggested differences by school level. Upper school students had a better understanding of the difference between gender roles and sex (item 1: RD 6.9%, 95%CI 0.8—13.1, *p* = 0.027; item 2: risk RD 9.4%, 95%CI 2.0-16.7, *p* = 0.012), sexual harassment (RD 11.8%, 95%CI 5.5—18.1, *p* < 0.001), rights and shared rights (RD 8.2%, 95%CI 1.7—14.7, *p* = 0.013), peer violence (RD 2.7%, 95%CI 1.6—3.8, *p* < 0.001) and policies (RD 14.9%, 95%CI 8.9—20.8, *p* < 0.001). There was a higher understanding of referrals to the student court among lower than upper school students (RD 12.7%, 95%CI 8.3—17.1, *p* < 0.001). There was also a higher understanding of sex vs. gender roles among those who had eaten zero meals or one meal yesterday (RD 2.9%, 95%CI −0.2—6.0) or two meals (RD 2.8%, 95%CI −2.3—7.8) compared to three or more meals (overall *p*-value = 0.008). Compared to students who had eaten three or more meals, there was a higher understanding of the student court for those who had eaten no meals or one meal (item 1: RD 5.6%, 95%CI −11.7—22.9; item 2: RD 14.5%, 95%CI 0.8—28.2) and lower understanding among those who had eaten two meals (item 1: RD 3.9%, 95%CI 0.9—7.0%) Overall *p*-values for item 1 and 2 were 0.003 and 0.024, respectively. Additional Files 3–5 show exploratory subgroup analyses for acceptability and understanding by sex, school level and number of meals eaten yesterday, respectively.

Among staff, 13 of 15 items assessing understanding were classified as green and two as red, of which both were related to the student court.

Results for individual items for acceptability and understanding constructs are presented in Table  6. 

**Table 6:** Acceptability and understanding of GST-S among staff and students

		Students		Staff	
Concept	Item	n agree*	% agree	n agree	% agree
**Acceptability**					
General acceptability and affective attitude	I like that our school is using the Good School Programme	430	95.8%	47	82.5%
	I would like our school to continue to implement activities of the Good School Programme	439	97.8%	49	86.0%
	I am likely to recommend the Good School Programme to another school	Not asked		51	89.5%
Burden	Participating in the Good School Programme was not a good use of my time *(%s represent % disagreeing)*	Not asked		49	86.0%
	I am willing to spend some of my time participating in the Good School Programme	445	99.1%	52	91.2%
Ethicality	It is important to me that our school is free from all violence	439	97.8%	53	93.0%
	Students should not be contributing to making decisions in school. It is only for teachers and staff to do this *(%s represent % disagreeing)*	398	88.6%	50	87.7%
Self-efficacy	I received enough training to take part in the Good School programme	Not asked		21	36.8%
	I feel confident to take part in the activities of the Good School Programme	437	97.3%	Not asked	
	I am able to access Good School materials when I need to	342	76.2%	31	54.4%
**Understanding**					
Sex/gender roles	_______ is what society expects a boy or girl to do *(fill in the blank)*^†^	401	89.3%	55	96.5%
	_______ is determined biologically which means it is how we are born *(fill in the blank)*^†^	355	79.1%	47	82.5%
Violence against children	Teachers, students and the school administration all have a responsibility to eliminate violence from schools	437	97.3%	57	100.0%
Positive discipline	A teacher considers how to respond to a student who is making noise in class and causing disruption. If the teacher asks the student to write an apology letter to the class, this would be an example of positive discipline.	440	98.0%	56	98.3%
Rights and shared rights	Students never have the responsibility to protect the rights of others	341	76.0%	51	89.5%
	Everyone at school has the same rights to physical safety, respect from others, to be listened to, and control over your body	435	96.9%	57	100.0%
Sexual harassment	Making unwanted sexual comments about a person is an example of sexual harassment	399	88.9%	54	94.7%
	It is not the responsibility of staff to protect students from sexual harassment	Not asked		56	98.3%
Peer violence	It is the whole school’s responsibility to stop students treating each other badly	435	96.9%	Not asked	
	If you see one of your friends bullying another student, it is best to pretend like you never saw it happen	435	96.9%	Not asked	
	There is no need for students who experience peer violence from a fellow student to go to a teacher for advice. They can sort it out between themselves.	Not asked		53	93.0%
	Providing life skills education to students can be helpful in preventing peer violence	Not asked		57	100.0%
	Teasing of students by their peers is not example of peer violence	Not asked		48	84.2%
Student court	Both students and teachers should make up the members of the student’s court	42	9.4%	11	19.3%
	Only teachers can refer cases of indiscipline to the student court	114	25.4%	4	7.0%
Policies	Only the school administration needs to be aware of school policies	343	76.4%	56	98.3%
	For a school to be managed effectively, it is important that the school has policies in place	432	96.2%	57	100.0%

*28 students across 3 schools were not asked these questions due to a survey routing error

†%s represent % giving correct answer

#### Fidelity

##### Fidelity progression criteria

The progression criterion for fidelity was green, with all schools implementing with fidelity. Two schools implemented eight markers, one school implemented seven markers and one school implemented six markers, as indicated in Table 7. All schools reported active teacher and student committees, and had implemented at least two activities. Data for step 3 markers were not available due to lack of documentation by schools; we instead used step 3 scores logged in the Raising Voices scorecard as proxy measures for the step 3 markers. The student court was not functional in any school and the wall of fame existed in three of the four schools. There was only one school which had a functional suggestion box appropriately used by teachers and students. All schools had a sustainability plan in place.

**Table 7:** Implementation of core fidelity markers

School		1	2	3	4
Proposed structures/activities for fidelity markers for each step					
**Step one: Your team and network**					
Teachers’ committee		Active	Active	Somewhat active	Active
Students’ committee		Active	Active	Active	Active
Parents’ committee		Active by step 4 (not step 2)	Not active	Initially somewhat active. Not active by step 4.	Active
**Step two: Preparing for change**					
Implemented at least 2 activities		Yes	Yes	Yes	Yes
Held committee meeting at least once a month​		No (only one recorded in activity log)	Unclear	Unclear	Unclear
**Step three: Good teachers and teaching**					
Student-teacher interaction		Teachers and students do not interact in friendly way	Friendly and respectful interaction between teachers and students	Teachers and students do not interact in friendly way	Friendly and respectful interaction between teachers and students
Student-student interaction		Somewhat friendly interaction	Somewhat friendly interaction	Somewhat friendly interaction	Somewhat friendly interaction
**Step four: Positive discipline**					
Students’ court in place		Exists but not functional	No	No	No
Wall of fame in place		Yes	Yes	No	Yes
**Step five: Good learning environment**					
Activities/structures promoting student’s voice e.g. functional suggestion boxes; existing policies promoting learners’ safety​		Suggestion box exists but is only used minimally due to its location next to the staff room	Suggestion box exists - used by teachers and students, and teachers review and feed back	Suggestion box exists and used but students feel teachers do not review or address suggestions	Suggestion box initially exists but not at second observation
**Step six: Administration and future**					
Sustainability plan in place​		Yes	Yes	Yes	Yes
**Total markers implemented**		7	8	6	8

The majority of data collected by Raising Voices was generally consistent with data from research team observations

Where inconsistent, data was discussed and resolved by both research team members and Raising Voices

##### Raising voices support visits

Raising Voices provided regular support visits to intervention schools, as shown in Table 8. Visits involved meetings with students, teachers, or parents. The purpose of meetings varied but included reviewing GST implementation progress, planning future activities, discussing methods to promote student voice and participation, training individuals on the GST-S concepts, and resolving other challenges arising.

**Table 8:** Raising voices support visits to schools

Content of session and number of participants														
School	1			2		3			4					
**Term 1 2022**														
Support visit 1	10 TPs across the four schools. Refresher training on key GST concepts.													
Support visit 2	Introducing GST-S concepts and key components													
	31 teachers 9 students			12 teachers 21 students		7 students			12 teachers, 17 students					
Support visit 3	Parents: Introducing GST-S concepts and role of parents committee. Students: Understanding role of students committee													
	11 parents 7 students			15 parents 11 students		15 parents 11 students			4 parents 19 students					
**Term 2 2022**														
Support visit 1	Teachers: understanding why children misbehave. Students: Reviewing and drawing step 3 action plans			Reviewing and drawing step 3 action plans		Understanding why children misbehave, using the discipline box. Additional review of progress with headteacher.			Teachers: understanding why children misbehave, step 3 action planning. Students: Reviewing and drawing step 3 action plans					
	24 teachers 8 students			2 teachers 26 students		12 teachers			16 teachers 4 students					
Support visit 2	N/A			N/A		Reviewing and drawing step 3 action plans			N/A					
						7 students								
**Term 3 2022**														
Support visit 1	Teachers and students: why voice matters *School 1– students additionally had content on smart choices.													
	15 teachers 54 students (with class not committee members)			12 teachers 15 students		10 teachers 8 students			11 teachers 14 students					
**Term 1 2023**														
Support visit 1	Corporal punishment and positive discipline alternatives													
	21 teachers 6 students			1 teacher 13 students		22 teachers 16 students			10 teachers 16 students					
Support visit 2	Review meeting to discuss lack of implementation progress			N/A		N/A			N/A					
	6 teachers (All teacher protagonists)													
**Term 2 2023**														
Support visit 1	Teachers: Positive discipline alternatives (request from students) Students: Life skills, mental health			Life skills, sustaining GST-S		Teachers: Positive discipline refresher Students: life skills			Teachers: student safety policies, sustaining GST-S, endline survey preparation. Students: life skills session					
	21 teachers 10 students			19 students		9 teachers 8 students			9 teachers 21 students					
Support visit 2	N/A			Positive discipline alternatives (delayed from first term)		N/A			N/A					
				46 teachers										

Data source: RV financial support records and activity log

Activities were done separately with teachers, students and parents unless specified otherwise

We were unable to determine the number of activities planned and implemented each term by schools. Schools did not provide action plans, which meant that we could not determine planned activities. Following implementation of an activity, teacher protagonists were supposed to complete an entry within an activity log. While some activities were recorded, there were several missing as confirmed by Raising Voices, and inconsistency between schools in the types and descriptions of activities recorded.

##### Individual-level exposure

For students in intervention schools, the mean score for exposure to GST-S (possible range 0–13) was 7.73 (SD = 3.45). Among staff, mean exposure was 7.35 (SD = 3.62). Scores were generally higher for questions related to passive participation in GST-S, such as seeing a GST-S poster, having a written classroom rules and having a suggestion box, compared to scores related to active participation, such as participating in an activity organised by the students committee or a discussion about GST-S posters or booklets. Results for individual-level exposure are included in Additional File 6.

### Exploratory analysis for phase 3 outcomes

A secondary outcome of this pilot trial was to explore preliminary estimates for the prevalence and clustering of phase 3 trial outcomes (sexual, physical and emotional violence from school staff, peers and intimate partners). It is important to note that this pilot trial was not powered to detect differences by arm in these outcomes, and we were also unable to fully adjust for baseline differences between schools due to our disrupted data baseline collection. However, for completeness we report confidence intervals from the exploratory analysis of phase 3 trial outcomes, and the ICC for outcomes for control schools at endline, in Additional File 7.

## Discussion

### Main findings

Our primary aim was to determine whether progression to a phase 3 cluster randomised trial is justified in terms of prespecified acceptability and feasibility criteria. According to our traffic light system, with the exception of one amber criterion, all criteria were categorised as green, suggesting that progression is justified. The intervention was acceptable and understandable to students and staff, and delivered with fidelity. The research was also feasible to conduct, with the criterion for enrolment classified as amber, and criteria for randomisation, follow-up and response rate as green.

#### Is the GST-S understandable and acceptable?

In relation to specific objectives, our first objective was to refine and finalise the GST-S intervention, exploring whether GST-S was acceptable and understandable to all students and staff. Staff and students reported high overall acceptability of GST-S, with few differences by sex, school level or socio-economic status of students. Individuals had positive attitudes towards GST-S, accepted the burden of participation, and were aligned with GST-S principles. However, staff and students reported difficulties in accessing GST-S materials. The quantities of materials provided was not proportionate to size of school populations, and the smallest school was the only one to not report difficulties with access.

Staff also reported that they received insufficient training. This may be because of disinterest, or lack of availability. It may also be that staff interpreted ‘training’ in the survey question as external training rather than the bite-size in-school training which was delivered in GST-S. However, it is more common for staff in secondary compared to primary schools to work part-time or move between schools, which may make it difficult for staff to participate in all trainings, and to train other staff within their school [38, 39]. It is important to address both training and access to materials given their contribution to the sustainability of public health interventions in schools [40], but these are resolvable issues and can be refined prior to a phase 3 trial.

The high acceptability and understanding among both female and male students is promising given that girls at primary school level reported reduced exposure to GST-P compared to boys [41]. While there was high understanding of GST-S generally, the student court was an exception to this. Monitoring data suggested that no schools had a functional student court in place, which may explain the poor understanding. Upper-school students had better understanding of some concepts, including sexual harassment, sex and gender roles, peer violence and school policies. This is unsurprising given that students are older, and may also be explained by the fact that committees were made up of more upper school students. However, our results also suggest that the intervention could potentially benefit from considering how to communicate ideas in a way to better support the learning and understanding of younger students, and encourage younger students to be part of GST-S committees.

#### Is it feasible to deliver GST-S with fidelity?

Our second objective was to understand the feasibility of delivering GST-S. Implementation was conducted over 18 months, but was slower than planned due to Covid-19, Ebola and consequent school closures. The extensive closures meant that schools were busier than usual and had many competing priorities after reopening. There was also a new curriculum in February 2020, which created additional pressure on schools. Despite this, all schools implemented GST-S with fidelity.

The student court was the marker implemented with the poorest fidelity. This may be explained by the timing of implementation, with condensed academic schedules limiting time to make use of this structure. Implementers also suggested that some schools had existing disciplinary structures in place with alternative names or that did not align with the student court. Some teachers also resisted the idea of a student court, which we hope our qualitative data will further explain.

The lack of action plans and log of activities recorded by teacher protagonists meant we had to change our approach to describing fidelity of implementation. Similar issues were also observed in the primary school trial, where records were also incomplete [20]. We suspect that this is likely explained by problems with documentation rather than implementation, given the positive findings from the scorecard data and expert opinion from implementers at Raising Voices. This is also reinforced by the survey results for individual-level exposure, which did show awareness and participation in GST-S.

However, scores for fidelity for staff and students in secondary schools were lower than what we observed in our primary school trial [20], possibly due to increased activity and pressures in secondary schools. We note lower scores in secondary versus primary schools for active participation in GST-S compared to passive exposure. We also noted low reported exposure to mental health which is an important new concept in GST-S compared to GST-P.

#### Is it possible to conduct a phase 3 trial?

Our third objective was to explore design parameters for a subsequent phase 3 trial, and we did note some important differences between secondary schools and our previous experience in primary schools. In terms of school enrolment, only five of the original schools selected participated in the baseline survey. The main reasons for schools not participating were busy schedules and complex administrative requirements for approval for participation. This was in contrast to our primary schools trial, where 100% of schools approached agreed to participate and recruitment was more straightforward. While our results suggest it is certainly possible to recruit secondary schools into a trial, they also suggest that secondary schools are busier and have more complex administrative requirements, which may be a barrier to participation for many schools. The research team noted that having a representative from Raising Voices at recruitment meetings with schools was effective in handling questions raised about GST-S and simplified the recruitment process. Once schools agreed to participate, attrition was low with all seven schools that participated in baseline remaining in the trial throughout. Student and staff participation rates were high.

### Strengths and limitations

Only one school declined to participate after consenting and we had high student and teacher response rates at endline. However, there were several disruptions to the trial due to the Covid-19 pandemic and Ebola outbreak, which led to a large amount of missing baseline data. It may have also affected engagement of schools with the programme due to schools’ competing priorities, and it meant that we were unable to reliably estimate our indicative trial outcomes at baseline.

Given that this is a pilot trial, a small number of schools were included, but we recruited schools in a stratified manner to capture important sources of variation in implementation that would be relevant to a phase 3 trial. However, we may have missed additional factors that are important in influencing implementation. Schools were only in Kampala and Wakiso districts, and it is important for a future trial to consider differences in implementation and research feasibility in other districts in Uganda.

There is likely some social desirability bias in survey responses, particularly for questions measuring acceptability and understanding of GST-S. However, scores for acceptability and understanding were not uniformly positive, suggesting that these questions do discriminate between high and low scores.

We were unable to blind participants to treatment allocation, a common challenge in behavioural intervention studies, but we measured violence using self-reported experiences, which is the gold standard in violence research. While this may be influenced by social desirability bias and a response shift bias [42], we intend to use student self-reported experiences rather than staff reports of use of violence as the primary phase 3 trial outcome, given that students reports are likely to be biased in the opposite direction to the intervention effect.

It is important to note that scorecard data was only collected twice in 2022 and there may have been some changes to fidelity markers following the second collection of scorecard data up until the end of implementation in either a positive or negative direction.

We did not ask participants about previous exposure to GST-P. While there is a possibility that staff and students had prior exposure to GST-P, this is unlikely to have affected many participants and therefore results.

### Implications

The pilot trial presents the first evidence that it is feasible to deliver a whole-school intervention that aims to address multiple forms of violence against adolescents from multiple perpetrators in sub-Saharan Africa. The intervention was well-delivered given the context of the Covid-19 pandemic and Ebola outbreak. It was highly acceptable and understandable to students and staff. Schools implemented most core structures and activities deemed essential by Raising Voices. Our results therefore suggest that it is appropriate to progress to a phase 3 trial.

While we cannot assume the results are generalisable to secondary schools across Uganda given the small sample size, we included a range of types of schools, and the promising results highlight the value of progressing to a phase 3 trial, particularly given the challenging circumstances in which this trial was conducted.

#### Recommendations for phase 3 trial

While we recommend progression, we propose several refinements to the research methods of a phase 3 trial. There were some challenges in recruitment of schools and initial agreement for participation. Given that this affected both government and private, and urban and rural schools, it may not be specific to certain types of schools, but having a representative from Raising Voices present throughout engagement with schools may improve recruitment. There were also challenges to measuring fidelity due to the lack of documentation by schools. Qualitative data may provide more insight into why this was the case. In a phase 3 trial, we will also consider alternative approaches to measuring fidelity, including more frequent collection of in-depth monitoring data, and finding novel ways to encourage schools to document GST-S progress.

Our findings also point to possible refinements to the intervention. We recommend ensuring equitable access to sufficient materials for all students and staff. Further information is needed on the materials required for secondary schools of different sizes and how materials are being used, but close attention should be paid to how materials are accessed, by whom and how often. Qualitative data will provide insight into which materials are most helpful and lacking for students and staff.

Attention also needs to be paid towards the approaches used for lower- vs. upper-school students. While GST-S materials and ideas were developed for adolescents, the range in age groups across secondary school students may mean that understanding of concepts differs by age. Additional emphasis may also be needed on equitable leadership across lower and upper secondary to ensure students of all ages participate in committees and GST-S leadership. We also need further reflection of how similar topics might be tailored towards different school levels, whether concepts need to be communicated differently to upper and lower school students and whether topics may align within the current curriculum.

To address the insufficient training received by staff, further exploration is needed to understand how best to structure training to ensure it is accessible by as many teachers as possible, as simply providing more training may not solve the issues around availability and turnover of staff. Given the poor implementation and resistance around the student court, and competing pressures within secondary schools, we recommend harmonising GST-S structures and activities with those already existing in schools where possible.

## Conclusion

This study provides the first evidence of the feasibility and acceptability of delivering a whole-school intervention to reduce multiple forms of violence against adolescents in sub-Saharan Africa. Based on our results, we recommend progression to a phase 3 trial with minor refinements to the research methods and intervention. Findings from qualitative data will provide further insight into the acceptability, understanding and fidelity of delivering GST-S, but these initial findings highlight the value of conducting a large-scale trial to evaluate the effectiveness of delivering GST-S.

## Supplementary Information

Additional file 1. CONSORT checklist (.doc). CONSORT 2010 checklist for the GST-S pilot trial.

Additional file 2. Overview of the adapted GST-S content (.doc). Table describing the original, strengthened and new content in GST-S compared to GST-P.

Additional file 3. Subgroup analysis of acceptability and understanding among students by sex (.doc). Results for the exploratory subgroup analysis of acceptability and understanding by sex.

Additional file 4. Subgroup analysis of acceptability and understanding among students by school level (.doc). Results for the exploratory subgroup analysis of acceptability and understanding by lower vs. upper school.

Additional file 5. Subgroup analysis of acceptability and understanding by number of meals (.doc). Results for the exploratory subgroup analysis of acceptability and understanding by number of meals eaten yesterday.

Additional file 6. Individual-level exposure to GST-S. Results for self-reported exposure to GST-S among students and staff.

Additional file 7. Exploratory analysis of phase 3 trial outcomes.

## Data Availability

The datasets used and/or analyzed during the current study will be made available from the corresponding author upon reasonable request.
