# Supplementary material for: The Good School Toolkit–Secondary to prevent violence against students: a pilot cluster randomised controlled trial
Source: BMC Public Health. 2025 Nov 6;25:3802. doi: 10.1186/s12889-025-23913-8 (PMC12590610; doi:10.1186/s12889-025-23913-8)
Supplement: Supplementary file 2 — Additional file 2. Overview of the adapted GST-S content (.doc). Table describing the original, strengthened and new content in GST-S compared to GST-P. [file 12889_2025_23913_MOESM2_ESM.docx]

**Additional file 2: Overview of the adapted GST-S content**

| **Step** | **School-led Activities** | **Content of Leadership Workshop Modules** |
| --- | --- | --- |
| **STEP 1 - Creating Team/Network** | 1.1 Good School Network  **1.2 Admin* Introduces GST to School**  **1.3 Recruit Teachers to GSC**  **1.4 Recruit Students to GSC**  **1.5 Recruit Community Members to GSC**  1.6 Recruit Admin* to GSC  **1.7 Subcommittee Welcome Meetings**  **1.8 Leadership Workshop 1: GSC Training (WS 1.1-1.8)**  1.9 Good School Morning 1: Our Shared Rights (WS 1.6) | **1.1 What Is a Good School?**  1.2 Creating a Conducive Learning Environment  1.3 What Is a Good Teacher?  1.4 Creating Positive Discipline at Your School  **1.5 What Is Good Governance?**  1.7 Four Types of Leaders  **1.8 Using Participatory Facilitation** |
| **STEP 2 - Preparing for Change** | 2.1 Create Plan  **2.2 Survey**  2.3 Bulletin Board  **2.4 Leadership Workshop 2: GSC Training (WS 2.1-2.7)**  **2.5 School-Wide Initiatives and Activities**  2.6 Good School Morning 2: Four Types of Leaders (WS 1.7)  2.7 One-Week Power Campaign  2.8 Launch GST | 2.1 Our shared rights  **2.2How are you using your power**  **2.3Types of Violence**  2.4 Peer Violence  2.5 Gender in Schools  **2.6 Challenging Gender Roles**  **2.7 Sexual Violence in Schools**  **2.8 Revisiting Participatory Facilitation** |
| **STEP 3 - Good Teachers/Teaching** | 3.1 Create Plan  **3.2 Leadership Workshop 3: School Staff (WS 3.1-3.8)**  **3.3 Student-Teacher Relationships**  **3.4 Creative Teaching**  ~~3.5 Bi-Monthly Teacher Meetings~~  **3.6 Professional Goals & Feedback**  3.7 Good School Morning 3: Gender in Schools (WS 2.4)  3.8 Gender Campaign | **3.1 Remembering Relationships**  3.2 Professional Pride  3.3 Challenging Gender Roles  3.4 Teaching for Both Genders  **3.5 Creating Teaching Techniques**  3.6 Why Do Students Misbehave?  3.7 Being a Role Model  3.8 Why Go to a Good School? (Peer Pressure) |
| **STEP 4 - Positive Discipline** | 4.1 Create Plan  4.2 Leadership Workshop 4: School Staff (WS 4.1-4.7)  4.3 Reinforce Positive Discipline Commitment  4.4 Recognize Student Strengths  4.5 Classroom Rules  4.6 Student Court  **4.7 School Standards and Rules**  4.8 Good School Morning 4: Peer Violence (WS 2.3)  4.9 Peer Violence Campaign | 4.1 What Is Corporal Punishment?  4.2 Corporal Punishment on Trial  4.3 Punishment vs. Discipline  4.4 Why Voice Matters  4.5 Positive Discipline Responses  4.6 Positive Discipline Role-Role Play  4.7 Encouraging Good Behavior |
| **STEP 5 - Good Learning Environ-ment** | 5.1 Create Plan  **5.2 Create Code of Conduct**  5.3 Share Code of Conduct  5.4 Student Leadership Opportunities  5.5 Prepare Students for Leadership (peer to peer)  5.6 Create a Student Referral Directory  5.7 Engage the community in caring for the Physical Compound  5.8 Good School Morning 5: Sexual Violence in Schools (WS 2.6)  5.9 Good School Parent’s Day | 5.1 Smart choices  5.2 Address sexual violence in school  5.3 Team building and cooperation  5.4 Respect and responsibility  5.5 Self-esteem and value  5.6 Friendship and relationship  5.7 Gender and self image |
| **STEP 6 - School Governance/ Way Forward** | 6.1 Create Plan  6.2 Good School Morning 6: Why go to a Good School? (WS 3.8)  6.3 Good School Assessment  6.4 Defining Way the Forward  6.5 Transition Meeting  6.6 Community Celebration |  |
| *Note.* The original or unchanged Good School Toolkit content in plain text, the strengthened content in **bold text** and the new content underlined. GSC is Good School Committee, GST-Good School Toolkit, Admin-school administration, WS-Workshop. | | |
| Previously published in GST-S protocol^1^ | | |

**References**

1. Devries K, Tanton C, Knight L, Nakuti J, Nanyunja B, Laruni Y, et al. Good School Toolkit-Secondary Schools to prevent violence against students: protocol for a pilot cluster randomised controlled trial. BMJ Open [Internet]. 2024 Feb 1;14(2):e077788. Available from: http://bmjopen.bmj.com/content/14/2/e077788.abstract
