# Supplementary material for: The Good School Toolkit–Secondary to prevent violence against students: a pilot cluster randomised controlled trial
Source: BMC Public Health. 2025 Nov 6;25:3802. doi: 10.1186/s12889-025-23913-8 (PMC12590610; doi:10.1186/s12889-025-23913-8)
Supplement: Supplementary file 3 — Additional file 3. Subgroup analysis of acceptability and understanding among students by sex (.doc). Results for the exploratory subgroup analysis of acceptability and understanding by sex. [file 12889_2025_23913_MOESM3_ESM.docx]

**Additional file 3. Subgroup analysis of acceptability and understanding among students by sex**

|  | |  |  |  | | | |  |  | |  |  |  |  |
| --- | --- | --- | --- | --- | --- | --- | --- | --- | --- | --- | --- | --- | --- | --- |
| **Concept** | | **Item** | **Male** | |  | **Female** | | **RD** | | **95%CI** | | | **p-value** |  |
|  | |  | **n agree** | | **% agree** | **n agree** | **% agree** |  | |  | |  |  |  |
| **Acceptability** | |  |  | |  |  |  |  | |  | |  |  | |
| General acceptability and affective attitude | | I like that our school is using the Good School Programme | 210 | | 94.2% | 220 | 97.4% | 3.2% | | -1.3% | | 7.6% | 0.163 | |
|  |  | I would like our school to continue to implement activities of the Good School Programme | 215 | | 96.4% | 224 | 99.1% | 2.7% | | -1.8% | | 7.2% | 0.241 | |
|  |  | I am willing to spend some of my time participating in the Good School Programme | 220 | | 98.7% | 225 | 99.6% | 0.9% | | -0.2% | | 2.0% | 0.116 | |
| Ethicality | | It is important to me that our school is free from all violence | 218 | | 97.8% | 221 | 97.8% | 0.0% | | -1.8% | | 1.8% | 0.974 | |
|  |  | Students should not be contributing to making decisions in school. It is only for teachers and staff to do this* | 198 | | 88.8% | 200 | 88.5% | -0.3% | | -8.0% | | 7.5% | 0.941 | |
|  |  | I feel confident to take part in the activities of the Good School Programme | 216 | | 96.9% | 221 | 97.8% | 0.9% | | -4.9% | | 6.7% | 0.753 | |
|  |  | I am able to access Good School materials when I need to | 164 | | 73.5% | 178 | 78.8% | 5.2% | | -3.9% | | 14.4% | 0.264 | |
| **Understanding** | |  |  | |  |  |  |  | |  | |  |  | |
| Sex/gender roles | | _______ is what society expects a boy or girl to do *(fill in the blank)**** | 199 | | 89.2% | 202 | 89.4% | 0.1% | | -8.6% | | 8.9% | 0.974 | |
|  |  | _______ is determined biologically which means it is how we are born *(fill in the blank)**** | 174 | | 78.0% | 181 | 80.1% | 2.1% | | -11.3% | | 15.4% | 0.763 | |
| Violence against children | | Teachers, students and the school administration all have a responsibility to eliminate violence from schools | 215 | | 96.4% | 222 | 98.2% | 1.8% | | -3.2% | | 6.9% | 0.479 | |
| Positive discipline | | A teacher considers how to respond to a student who is making noise in class and causing disruption. If the teacher asks the student to write an apology letter to the class, this would be an example of positive discipline. | 219 | | 98.2% | 221 | 97.8% | -0.4% | | -3.6% | | 2.8% | 0.797 | |
| Rights and shared rights | | Students never have the responsibility to protect the rights of others* | 163 | | 73.1% | 178 | 78.8% | 5.7% | | 0.0% | | 11.3% | 0.05 | |
|  |  | Everyone at school has the same rights to physical safety, respect from others, to be listened to, and control over your body | 213 | | 95.5% | 222 | 98.2% | 2.7% | | 0.7% | | 4.7% | 0.008 | |
| Sexual harassment | | Making unwanted sexual comments about a person is an example of sexual harassment | 196 | | 87.9% | 203 | 89.8% | 1.9% | | -1.9% | | 5.7% | 0.32 | |
| Peer violence | | It is the whole school’s responsibility to stop students treating each other badly | 211 | | 94.6% | 224 | 99.1% | 4.5% | | -0.8% | | 9.8% | 0.097 | |
|  |  | If you see one of your friends bullying another student, it is best to pretend like you never saw it happen | 217 | | 97.3% | 218 | 96.5% | -0.8% | | -4.1% | | 2.4% | 0.608 | |
| Student court | | Both students and teachers should make up the members of the student’s court | 24 | | 10.8% | 18 | 8.0% | -2.8% | | -5.8% | | 0.2% | 0.065 | |
|  |  | Only teachers can refer cases of indiscipline to the student court | 57 | | 25.6% | 57 | 25.2% | -0.3% | | -3.9% | | 3.2% | 0.850 | |
| Policies | | Only the school administration needs to be aware of school policies | 174 | | 78.0% | 169 | 74.8% | -3.2% | | -11.5% | | 5.0% | 0.439 | |
|  |  | For a school to be managed effectively, it is important that the school has policies in place | 216 | | 96.9% | 216 | 95.6% | -1.3% | | -3.2% | | 0.6% | 0.192 | |
| * Reverse coded and %s represents % disagreeing/strongly disagreeing.  ‡ %s represent % giving correct answer  Abbreviations: CI, confidence interval; RD, risk difference  28 students across 3 schools were not asked these questions due to a survey routing error and were excluded from the analysis. A small proportion of students responded ‘Don’t know’ or declined to respond across 9 of the items (<3% for all 9 items). These individuals were coded as the response option reflecting low acceptability or understanding to provide the most conservative.  Analyses are adjusted for clustering by school | | | | | | | | | | | | | | |
