# Supplementary material for: The Good School Toolkit–Secondary to prevent violence against students: a pilot cluster randomised controlled trial
Source: BMC Public Health. 2025 Nov 6;25:3802. doi: 10.1186/s12889-025-23913-8 (PMC12590610; doi:10.1186/s12889-025-23913-8)
Supplement: Supplementary file 6 — Additional file 6. Individual-level exposure to GST-S. Results for self-reported exposure to GST-S among students and staff. [file 12889_2025_23913_MOESM6_ESM.docx]

**Additional file 6. Individual-level exposure to GST-S**

|  | **Students*** | | | | | | **Staff** | | | | | |
| --- | --- | --- | --- | --- | --- | --- | --- | --- | --- | --- | --- | --- |
|  | **Control** | | **Intervention** | | **All** | | **Control** | | **Intervention** | | **All** | |
|  | n | % | n | % | n | % | n | % | n | % | n | % |
| Overall exposure score (0-13), mean (SD) | 4.43 | 3.27 | 7.73 | 3.45 | 6.31 | 3.75 | 2.63 | 2.46 | 7.35 | 3.62 | 5.38 | 3.94 |
| **Individual items** |  | |  | |  | |  | |  | |  | |
| My school has a students’ court that is different than the prefects council or discipline committee | 75 | 20.8% | 225 | 47.2% | 300 | 35.8% | 9 | 22.0% | 24 | 42.1% | 33 | 33.7% |
| My school has a Good Schools students committee | 129 | 35.8% | 360 | 75.5% | 489 | 58.4% | 8 | 19.5% | 42 | 73.7% | 50 | 51.0% |
| I have participated in an activity organised by the Good Schools students committee | 104 | 28.9% | 211 | 44.2% | 315 | 37.6% | 5 | 12.2% | 29 | 50.9% | 34 | 34.7% |
| My school has written classroom rules and regulations for how students should be | 247 | 68.6% | 374 | 78.4% | 621 | 74.2% | 12 | 29.3% | 39 | 68.4% | 51 | 52.0% |
| My class participated in making up these rules | 131 | 36.4% | 280 | 58.7% | 411 | 49.1% | 5 | 12.2% | 26 | 45.6% | 31 | 31.6% |
| These written rules are displayed in my classroom where students can see them | 115 | 31.9% | 258 | 54.1% | 373 | 44.6% | 9 | 22.0% | 25 | 43.9% | 34 | 34.7% |
| My school has a wall of fame for students | 85 | 23.6% | 285 | 59.8% | 370 | 44.2% | 6 | 14.6% | 32 | 56.1% | 38 | 38.8% |
| My school has a suggestion box where students can put ideas | 288 | 80.0% | 408 | 85.5% | 696 | 83.2% | 31 | 75.6% | 54 | 94.7% | 85 | 86.7% |
| In my school, I have seen a poster or booklet about Good Schools, like this (*image of material*) | 89 | 24.7% | 375 | 78.6% | 464 | 55.4% | 9 | 22.0% | 46 | 80.7% | 55 | 56.1% |
| In my school, I have participated in a discussion about Good Schools posters or booklets | 46 | 12.8% | 168 | 35.2% | 214 | 25.6% | 3 | 7.3% | 26 | 45.6% | 29 | 29.6% |
| My school has a Good Schools teacher committee | 126 | 35.0% | 313 | 65.6% | 439 | 52.5% | 3 | 7.3% | 35 | 61.4% | 38 | 38.8% |
| My school has a Good Schools parents committee | 97 | 26.9% | 187 | 39.2% | 284 | 33.9% | 3 | 7.3% | 21 | 36.8% | 24 | 24.5% |
| I have seen a poster on mental health from the Good Schools programme | 64 | 17.8% | 244 | 51.2% | 308 | 36.8% | 5 | 12.2% | 20 | 35.1% | 25 | 25.5% |

*Missing data from 39 students in control arm and 28 students in intervention arm due to a survey routing error.
