# Supplementary material for: The Good School Toolkit–Secondary to prevent violence against students: a pilot cluster randomised controlled trial
Source: BMC Public Health. 2025 Nov 6;25:3802. doi: 10.1186/s12889-025-23913-8 (PMC12590610; doi:10.1186/s12889-025-23913-8)
Supplement: Supplementary file 7 — Additional file 7. Exploratory analysis of phase 3 trial outcomes. [file 12889_2025_23913_MOESM7_ESM.docx]

**Additional file 7. Exploratory analysis of phase III trial outcomes**

|  |  | **Prevalence** | | | | | | **Comparison between control and intervention** | | | | | |
| --- | --- | --- | --- | --- | --- | --- | --- | --- | --- | --- | --- | --- | --- |
|  |  | **Control** | | | **Intervention** | | | **95% CI for unadjusted OR** | | | **95% CI for adjusted OR** | | |
|  |  | **n** | **%** | | **n** | | **%** |  | | |  | | |
| **Past week violence** | |  | | | | | |  | | |  | | |
| Physical violence | |  | |  | |  |  |  | |  |  |  | |
|  | From school staff | 109 | | 30.3% | | 210 | 44.0% | 1.04 | | 3.14 | 0.30 | 71.30 | |
|  | From peers | 39 | | 10.8% | | 56 | 11.7% | 0.59 | | 2.01 | 0.07 | 0.80 | |
| Sexual violence | |  | |  | |  |  |  | |  |  |  | |
|  | From school staff | 9 | | 2.5% | | 15 | 3.1% | 0.55 | | 2.93 | 0.28 | 0.98 | |
|  | From peers | 22 | | 6.1% | | 20 | 4.2% | 0.23 | | 1.77 | 0.02 | 0.51 | |
| Emotional violence | |  | |  | |  |  |  | |  |  |  | |
|  | From school staff | 43 | | 11.9% | | 86 | 18.0% | 0.90 | | 2.83 | 3.39 | 110.03 | |
|  | From peers | 115 | | 31.9% | | 179 | 37.5% | 0.96 | | 1.71 | 0.73 | 1.62 | |
| **Past term violence** | |  | | | | | |  |  | |  | |  |
| Physical violence | |  | |  | |  |  |  | |  |  |  | |
|  | From school staff | 229 | | 63.6% | | 333 | 69.8% | 0.89 | | 1.99 | 0.54 | 26.51 | |
|  | From peers | 91 | | 25.3% | | 104 | 21.8% | 0.56 | | 1.22 | 0.02 | 0.78 | |
|  | From intimate partners | 2 | | 0.6% | | 1 | 0.2% | 0.03 | | 4.16 | N/A |  | |
| Sexual violence | |  | |  | |  |  |  | |  |  |  | |
|  | From school staff | 30 | | 8.3% | | 45 | 9.4% | 0.71 | | 1.86 | 0.06 | 87.73 | |
|  | From peers | 51 | | 14.2% | | 53 | 11.1% | 0.50 | | 1.14 | 0.06 | 2.27 | |
|  | From intimate partners | 5 | | 1.4% | | 7 | 1.5% | 0.33 | | 3.36 | 0.04 | 4.45 | |
| Emotional violence | |  | |  | |  |  |  | |  |  |  | |
|  | From school staff | 137 | | 38.1% | | 197 | 41.3% | 0.84 | | 1.56 | 0.07 | 1.24 | |
|  | From peers | 333 | | 69.8% | | 234 | 65.0% | 0.93 | | 1.67 | 0.11 | 5.38 | |
|  | From intimate partners | 22 | | 6.1% | | 35 | 7.3% | 0.70 | | 2.11 | 0.09 | 29.02 | |
| *Adjusted for sex, assignment to ACASI or FTFI group, urban/rural, faith/non-faith based, and school-level baseline value of outcome (or mean baseline value of outcome for arm if missing at baseline).  Abbreviations: CI, confidence interval; OR, odds ratio  The ICCs for past week physical, sexual and emotional violence from school staff were 2.43e-23, 1.3e-14 and 2.85e-21, respectively. The ICCs for past week physical, sexual and emotional violence from peers were 0.03, 0.06, 0.01, respectively. The ICCs for past term physical, sexual and emotional violence from intimate partners were 4.83e-24,1.42e-19, 3.00e-24. The ICC was calculated at endline from control schools. | | | | | | | | | | | | | |
